# Food restriction affects maternal investment but not neonate phenotypes in a viviparous lizard

**DOI:** 10.24272/j.issn.2095-8137.2017.011

**Published:** 2017-03-18

**Authors:** Yang Wang, Zhi-Gao Zeng, Liang Ma, Shu-Ran Li, Wei-Guo Du

**Affiliations:** ^1^Key Laboratory of Animal Ecology and Conservational Biology, Institute of Zoology, Chinese Academy of Sciences, Beijing 100101, China; ^2^University of Chinese Academy of Sciences, Beijing 100049, China

**Keywords:** *Eremias multiocellata*, Food availability, Hatchling, Lizard, Reproductive output

## Abstract

Food availability significantly affects an animal's energy metabolism, and thus its phenotype, survival, and reproduction. Maternal and offspring responses to food conditions are critical for understanding population dynamics and life-history evolution of a species. In this study, we conducted food manipulation experiments in field enclosures to identify the effect of food restriction on female reproductive traits and postpartum body condition, as well as on hatchling phenotypes, in a lacertid viviparous lizard from the Inner Mongolian desert steppe of China. Females under low-food availability treatment (LFT) had poorer immune function and body condition compared with those under high-food availability treatment (HFT). The food availability treatments significantly affected the litter size and litter mass of the females, but not their gestation period in captivity or brood success, or the body size, sprint speed, and sex ratio of the neonates. Females from the LFT group had smaller litter sizes and, therefore, lower litter mass than those from the HFT group. These results suggest that female racerunners facing food restriction lay fewer offspring with unchanged body size and locomotor performance, and incur a cost in the form of poor postpartum body condition and immune function. The flexibility of maternal responses to variable food availability represents an important life strategy that could enhance the resistance of lizards to unpredictable environmental change.

## INTRODUCTION

In nature, the food available to an animal exhibits spatio-temporal variation, significantly affecting its energy metabolism and consequently its phenotype, survival, and reproduction (Acheampong et al., 2011; Douhard et al., 2014; Du, 2006; Hoy et al., 2016). Food availability not only affects maternal fitness-related traits (e.g. body size, growth, and reproduction), but might also induce phenotypic variations in offspring (Ballinger & Congdon, 1980; Hillesheim & Stearns, 1991; Jones et al., 2015; Vaissi & Sharifi, 2016). For example, food availability can lead to variations in maternal reproduction (Ballinger, 1977) and offspring growth and survival in lizards (Dunham, 1978; Warner et al., 2015). Therefore, maternal and offspring responses to food conditions are critical for understanding ecological and evolutionary processes, such as population dynamics and life-history evolution.

Food availability can significantly affect female reproductive strategies, such as reproductive timing, investment, and output (offspring number and size) (Ballinger, 1977; Du, 2006; Ramírez-Pinilla, 2006). Two kinds of trade-offs regarding energy allocation are faced by a mother when the energy available to her is limited. First, a mother has to decide on energy allocation for multiple tasks, such as maintenance, growth, and reproduction, leading to important life-history trade-offs (e.g., maintenance *vs.* reproduction) (Hegemann et al., 2013; Rollinson & Rowe, 2016); for instance, the tropical house wren (*Troglodytes aedon*) decreases parental reproductive investment (i.e. nestling feeding frequency), but does not alter self-maintenance (metabolic rate and body condition) when the cost of activity increases during reproduction (Tieleman et al., 2008). Second, a mother needs to decide on the distribution of energy among offspring within a clutch, thus leading to a trade-off between clutch size and offspring size (Bleu et al., 2013; Du et al., 2005; Olsson et al., 2002). The "optimal egg size theory" assumes that one optimal egg size is appropriate under certain maternal and environmental conditions to maximize the fitness of offspring (Einum & Fleming, 1999; Smith & Fretwell, 1974; Williams, 1966). Accordingly, a mother might give priority of energy allocation to offspring size rather than to clutch size when faced with food restriction, especially in those species whose fitness is determined primarily by egg size (Styrsky et al., 2000). However, other studies have shown that offspring size is not always optimized, and can be highly condition-dependent in some species (Krist & Munclinger, 2015; Rollinson & Hutchings, 2013), with the shift in size being a function of maternal reproductive investment (Caley et al. 2001). In such cases, a mother might change both offspring and clutch sizes in response to food restriction. Despite extensive studies regarding the effect of food availability on maternal reproductive traits (e.g. Bonnet et al., 2001; Du, 2006; Hogstedt, 1981; Kitaysky et al., 1999; Warner & Lovern, 2014; Warner et al., 2015), how female reproduction responds to food availability might differ among species and deserves further investigation.

In addition to maternal reproductive traits, food availability can also profoundly affect offspring phenotypes and fitness. Previous studies have shown that a variety of phenotypic traits of offspring (e.g., body size, locomotor performance, and growth rate) can be affected by maternal food availability (Du, 2006; Hafer et al., 2011; Raveh et al., 2016; Warner et al., 2015), likely through a maternal effect-a phenomenon in which environmental information passes through generations by means of plasticity rather than direct genetic transmission (Fox & Mousseau, 1998; Hsu et al., 2016); for example, low food availability compromises the snout-vent length (SVL) of offspring, as well as the mass, performance ability, and fat reserves of the viviparous lizard *Pseudemoia entrecasteauxii*(Itonaga et al., 2012b). Offspring sex is one of the most interesting phenotypes affected by maternal food availability. The "sex allocation hypothesis" suggests that females can manipulate the sex ratio of their offspring, contingent upon local conditions, to maximize offspring fitness (Rosenfeld & Roberts, 2004; Trivers & Willard, 1973). Food availability can affect the offspring sex ratio in several avian species; for example, when provided with supplementary food, the kakapo (*Strigops habroptilus*) female produces an excess of males (Clout et al., 2002). However, such studies are scarce for reptilian species (Wapstra & Warner, 2010; Warner et al., 2007).

Viviparous lizards make an excellent model for studying maternal and offspring responses to food availability because many lizards experience fluctuating food resources (Meserve et al., 2016; Zhu et al., 2014), and viviparous species retain their eggs for a longer period than oviparous species do, with higher locomotor and thermoregulatory costs (Le Galliard et al., 2003; Shine, 2003). Moreover, most ectothermic vertebrates are considered capital breeders, in which reproduction is financed from stored energetic capital (Bonnet et al.,1998), which makes the direct causal relationship between food availability and reproduction harder to detect. Conversely, many viviparous reptiles are considered income breeders because they can use maternal nutrients (matrotrophy) to supplement or replace yolk nutrients (lecithotrophy) for embryonic development (Bonnet et al.,2001; Ramírez-Pinilla, 2006; Winne et al., 2006). As a result, viviparous lizards may confront more severe trade-offs between reproduction and self-maintenance when experiencing limited resources during gestation, and thus provide a unique system to increase our understanding of the effects of food on life history strategies in animals. In this study, we conducted food manipulation experiments in field enclosures to identify the effect of food restriction on female reproductive traits (such as gestation period in captivity and litter size) and postpartum body condition, as well as hatchling phenotypes (body size and locomotor performance and immune response) in a lacertid viviparous lizard from the Inner Mongolian desert steppe of China. We aimed to address the following questions: (1) How does female reproductive investment respond to food restriction? (2) Does food restriction affect postpartum body condition of females? (3) How does maternal food restriction influence neonate phenotypes?

## MATERIALS AND METHODS

### Study species

The multi-ocellated racerunner (*Eremias multiocellata*) is a small viviparous lizard that inhabits desert or semiarid areas, with the SVL of adults ranging from 58 to 73 mm. This species is the main lizard fauna in our field study site at the Shierliancheng Field Station, Institute of Grassland Research of the Chinese Academy of Agricultural Sciences (Zeng et al., 2016), located in Ordos, Inner Mongolia, China (N40°12'17", E111°07'43"; elevation 1 036 m). Thermal and hydric environments significantly affect the reproductive traits and offspring phenotypes in different populations of this species, with the offspring sex ratio biased towards males when gravid females are kept at high temperatures (Tang et al., 2012; Wang et al., 2016).

### Maternal food treatment in field enclosures

Adult *E. multiocellata*(16 males and 48 females) were collected from our field study site and were housed in round enclosures (high×diameter=40 cm×180 cm) built at the field site (one male and three females in each enclosure) from May 20 to May 30, 2014, which is the beginning of the reproductive season for this species. All females had bitemarks on their belly, suggesting that they had mated in the field. The enclosures were covered with plastic nets to avoid predation by birds.

On June 1, 2014, the SVL and body mass of all females were measured, after which each female was randomly assigned to a treatment group with either high (*n*=24) or low (*n*=24) prey availability. Lizards in the high-food availability treatment (HFT) group were fed every other day with 0.05 g of mealworms per gram mass of female per day (amounting to 120% of the average consumption of a gravid female, as measured prior to the experiment), whereas those in the low-food availability treatment (LFT) group were fed at the same intervals with 0.025 g of mealworms per gram mass of female per day (amounting to 60% of the average consumption of a gravid female). Each treatment was replicated eight times (for a total of 16 enclosures). Given that all viviparous amniotes have a placenta that transfers maternal nutrients to the embryo (Flemming et al., 2003), viviparous *E. multiocellata* should be matrotrophic. The females of this species start to copulate in May, and give birth in July and August, with a gestation period of about 54 days (Tang et al., 2012). Our food treatments lasted 30 days (from June 1 to June 30), covering the middle half of the gestation period.

### Female reproductive traits and phenotypes of neonates

On July 1, 2014, females (16 females from HFT and 15 females from LFT) in the field enclosures were retrieved and transferred to the laboratory for measurement of reproductive traits. Females were maintained in small cages (long×wide×high=310×210×180 mm) with a substrate of sand and two small pieces of brick as shelter. The cages were exposed to the natural light regime of the field station, and a 60W incandescent light bulb was suspended 5 cm above each cage for thermoregulation from 0800 to 1200h. Food (mealworms and crickets dusted with additional vitamins and minerals) was provided daily *ad libitum*. Each cage contained two females and was checked once per day for neonates, and four times per day following first parturition.

Immediately after a female produced a litter of neonates, the mother was measured and weighed. The SVL and body mass of neonates were then measured to 0.01 mm and 0.001 g accuracy after the absorption of the yolks one day later. Litter size was determined as the number of neonates and litter mass was calculated as the total mass of neonates produced by a female (Li et al., 2006; Ramírez-Bautista et al., 2000). The gestation period in captivity was calculated as the days between the initiation of treatment and female parturition, which did not include the gestation period in the field before the females were collected. To measure the locomotor performance, each neonate was made to run on a racetrack (80 cm long, marked at 20-cm intervals), within 2-3 days of birth, by stimulation with a soft paintbrush (Irschick & Losos, 1998). This procedure was repeated twice at 30±1 ℃ allowing a break of 1 h between each test. For quantitating locomotor performance, the sprint speed was determined by averaging the fastest speeds for covering a distance of 20 cm in each race. The sex of neonates was identified by observing the preanal scales-males have large, square, regularly-distributed preanal scales, whereas females have small, round, and scattered preanal scales (Wang et al., 2016).

A total of 12 and 14 females produced offspring from the LFT and HFT groups, respectively. Offspring from four clutches in the LFT group and one clutch in the HFT group were stillborn and excluded from further analysis of locomotor performance and offspring sex. The brood success of females was calculated as the number of females producing live neonates / total number of females.

### Cellular immune response of postpartum females

Cellular immune response was assessed by administering an injection (20 μL) of 50 mg of phytohemagglutinin (PHA) in the right foot of postpartum *E. multiocellata* females. The thickness of the right and left feet were measured every 12 h from 0 to 36 h following the PHA injection. The difference in foot thickness was considered as an index of immune response (Brown et al., 2011; Vinkler et al., 2010). The largest difference in thickness (i.e., peak immune response) was found at 12 h. Thus, PHA-induced skin swelling at 12 h was used to identify the effect of food availability on the immune response of females.

### Statistical analysis

All analyses were performed with SPSS Statistics software (ver. 22; IBM Corp. 2014). Data were normalized by log-transformation when necessary. Maternal body condition was calculated as a residual score from the regression of body mass on SVL (Jakob et al., 1996). Treatment effects on maternal body condition, PHA response, reproductive output, and neonate traits were evaluated by one-way analysis of variance (ANOVA). Clutch means were calculated for neonate traits to avoid pseudo-replication. Pearson *Chi*-Square test was used for comparing between-treatment differences in brood success.

## RESULTS

**Figure 1:**
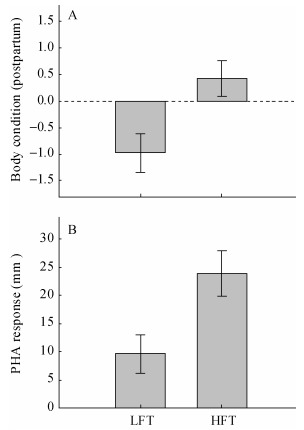
Effects of food availability treatment on female postpartum body condition (A) and immune function (B)

At the beginning of the experiment, both male and female body sizes did not differ between food treatments (Table 1). However, the postpartum body conditions were worse for females in the LFT group than for those in the HFT group (*F*_1, 24_=7.928, *P*=0.010) (Figure 1A), although initial body condition did not differ between the two food treatment groups (*F*_1, 24_=1.162, *P*=0.292). In addition, females from the LFT group had poorer immune function than those from the HFT group, as indicated by the lower PHA response of LFT group females (*F*_1, 23_=7.214, *P*=0.014, Figure 1B).

**Table 1:** Reproductive traits of *Eremias multiocellata* under high-and low-food availability treatment

Variable	Treatment		*P*
	Low food	High food	
**Adult body sizes**			
Male SVL (mm)	63.19±0.65 (*n*=8)	62.94±0.65 (*n*=8)	*F*_1, 14_=0.740, *P*=0.789
Male mass (g)	6.94±0.25 (*n*=8)	7.18±0.25 (*n*=8)	*F*_1, 14_=0.435, *P*=0.520
Female SVL (mm)	66.08±0.38 (*n*=24)	66.16±0.38 (*n*=24)	*F*_1, 46_=0.020, *P*=0.889
Female mass (g)	7.24±0.16 (*n*=24)	7.28±0.16 (*n*=24)	*F*_1, 46_=0.041, *P*=0.841
**Reproductive parameters**			
Brood success	53.33% (*n*=15)	81.25% (*n*=16)	*χ^2^*=1.631, *P*=0.202
Gestation period in captivity (days)	41.58±1.28 (*n*=12)	42.00±1.19 (*n*=14)	*F*_1, 24_=0.057, *P*=0.814
Litter size (*n*)	2.67±0.27 (*n*=12)	3.71±0.25 (*n*=14)	***F*_1, 24_=7.907, *P*=0.010**
Litter mass (g)	1.56±0.17 (*n*=12)	2.16±0.16 (*n*=14)	***F*_1, 24_=6.650, *P*=0.016**
**Neonate traits**			
Neonate SVL (mm)	30.15±0.33 (*n*=12)	29.52±0.30 (*n*=14)	*F*_1, 24_=1.991, *P*=0.171
Neonate mass (g)	0.59±0.02 (*n*=12)	0.58±0.02 (*n*=14)	*F*_1, 24_=0.008, *P*=0.930
Sprint speed (m/s)	0.85±0.13 (*n*=8)	0.95±0.10 (*n*=13)	*F*_1, 19_=0.355, *P*=0.558
Sex ratio (male%)	42.10±8.50 (*n*=8)	51.10±11.30 (*n*=13)	*F*_1, 19_=0.397, *P*=0.536

Values are expressed as means±*SE*. One-way ANOVA and Fisher's Exact Test were used to compare the between-treatment differences in reproductive traits. Brood success=number of females producing live neonates/total number of females. Neonate traits (except for sex ratio) were calculated as clutch means to avoid pseudo-replication. SVL, snout-vent length.

Food availability treatment did not affect brood success or gestation period in captivity of females, but did significantly affect both litter size and litter mass (Table 1). The females from the LFT group had smaller litter sizes and, therefore, lower litter mass compared with those for females from the HFT group (Table 1). Moreover, maternal food availability did not affect the body size (SVL and body mass), sprint speed, or sex ratio of the neonates (Table 1).

## DISCUSSION

When facing food restriction, female racerunners lay fewer offspring with unchanged body size and locomotor performance, but at a cost in terms of poor postpartum body condition and immune function. These maternal and offspring responses to food restriction have interesting implications for our understanding of the reproductive strategies of lizards under temporally fluctuating food abundance in nature.

A female must decide how to allocate limited resources to the processes of self-maintenance and reproduction (Du, 2006; Hegemann et al., 2013; Itonaga et al., 2012a). Obviously, female racerunners from the LFT group produced neonates with a similar body size to those produced by females from the HFT group, but at the cost of poor body condition and immune function. Understandably, lizards adopt a strategy of keeping reproductive investment relatively constant and sacrificing other requirements like those of immune function and energy storage under constrained food conditions, because maintaining a competent immune system is a nutritionally demanding process that necessitates trade-off decisions with competing nutrient demands, such as those of growth, reproduction, and thermal regulation (Sheldon & Verhulst, 1996; Uller et al., 2006; Zamora-Camacho et al., 2016). This strategy of females giving priority to reproduction rather than to self-maintenance has also been reported in other reptiles and vertebrate species; for example, the body condition of female jacky dragons (*Amphibolurus muricatus*) fed a low-quality diet declined dramatically throughout the reproductive season, whereas their hatchlings were larger and in better body condition than those produced from females fed a high-quality diet (Warner et al., 2007). Such a life strategy helps the reproduction and recruitment of populations, but may significantly weaken the immune response and, therefore, the survival of females via elevated risks of predation or starvation in their natural habitat (Iglesias-Carrasco et al., 2016; Neuman-Lee et al., 2015).

The pre-breeding nutritional condition of females is closely related to the production of neonates in oviparous vertebrates, with reduced fecundity under conditions of food limitation (Du, 2006; Donelson et al., 2008; Johnson et al., 2014; Lehman & Smith, 1988; Warner & Lovern, 2014). In response to decreased resources, the females of multiple-clutch species can reduce their reproductive output by decreasing litter size (Sun et al., 2002), offspring size (Abell, 1999), or reproductive frequency (Du, 2006). The viviparous racerunners in our study produced one litter per reproductive season, and decreased their litter size rather than offspring size in response to food restriction. This reproductive strategy reflects the highly critical reproductive investment for each hatchling, suggesting that an optimal neonate body size could exist independent of nutritional conditions. This is consistent with the optimal offspring size theory in which females reproducing in a given environment divide available resources into optimally-sized offspring (Sinervo & Licht, 1991; Smith & Fretwell, 1974). Nonetheless, further studies on the relationship between initial body size and fitness of neonates are needed to verify this hypothesis. At the same time, the reduction in clutch size under conditions of low food availability could be an effective strategy to improve offspring fitness. Fewer offspring means more abundant food per neonate in an environment with finite resources, which will reduce competition and maximize the survival and reproduction of descendants (Bartlett, 1988).

The traditional sex allocation theory generates two conflicting predictions on how maternal nutrition affects the offspring sex ratio (Hamilton, 1967; Trivers & Willard, 1973). Females may produce higher numbers of male offspring under low-food conditions because males might have higher fitness or are more likely to disperse under stressful food conditions (Komdeur et al., 1997; Warner et al., 2007). Alternatively, the opposite pattern may occur, because producing more daughters under low-food conditions will gain greater fitness return given that offspring fitness is less dependent on body size (hence, reproductive energy input) in daughters than in sons (Krist & Munclinger, 2015; Trivers & Willard, 1973). In the current case, however, females did not adjust the sex ratio of their offspring, giving no support to either prediction above.

Overall, female viviparous lizards may sacrifice their own health to produce high-quality offspring and maintain the sex ratio, which could maximize their reproductive success and individual fitness when experiencing low food availability. In addition to maternal and offspring responses to environmental factors like temperature and precipitation (Ma et al., 2014; Wang et al., 2016), the flexibility of maternal responses to variable food availability represents an important life strategy that might enhance the resistance of lizards to unpredictable environmental change. Understanding maternal and offspring responses to the combined impact of these biotic and abiotic factors is a considerable challenge and should be of great interest for future investigations.

## ACKNOWLEDGEMENTS

We thank Shao-Yong Chen and Zhi-Liang Jie for their assistance in the field and laboratory.

## References

[1] Abell AJ. 1999 Variation in clutch size and offspring size relative to environmental conditions in the lizard Sceloporus virgatus. Journal of Herpetology, 33 (2): 173- 180.

[2] Acheampong E, Campbell RW, Diekmann ABS, St John MA. 2011 Food availability effects on reproductive strategy:The case of *Acartia tonsa* (Copepoda:Calanoida). Marine Ecology Progress Series, 428 151- 159.

[3] Ballinger RE. 1977 Reproductive strategies:food availability as a source of proximal variation in a lizard. Ecology, 58 (3): 628- 635.

[4] Ballinger RE, Congdon JD. 1980 Food resource limitation of body growth rates in Sceloporus scalaris (Sauria, Iguanidae). Copeia, 1980 (4): 921- 923.

[5] Bartlett J. 1988 Male Mating success and paternal care in *Nicrophorus vespilloides* (Coleoptera, Silphidae). Behavioral Ecology and Sociobiology, 23 (5): 297- 303.

[6] Bleu J, Le Galliard JF, Fitze PS, Meylan S, Clobert J, Massot M. 2013 Reproductive allocation strategies:A long-term study on proximate factors and temporal adjustments in a viviparous lizard. Oecologia, 171 (1): 141- 151. 22791132 10.1007/s00442-012-2401-1

[7] Bonnet X, Bradshaw D, Shine R. 1998 Capital versus income breeding:An ectothermic perspective. Oikos, 83 (2): 333- 342.

[8] Bonnet X, Naulleau G, Shine R, Lourdais O. 2001 Short-term versus long-term effects of food intake on reproductive output in a viviparous snake, Vipera aspis. Oikos, 92 (2): 297- 308.

[9] Brown GP, Shilton CM, Shine R. 2011 Measuring amphibian immunocompetence:Validation of the phytohemagglutinin skin-swelling assay in the cane toad, *Rhinella marina*. Methods in Ecology and Evolution, 2 (4): 341- 348.

[10] Caley MJ, Schwarzkopf L, Shine R. 2001 Does total reproductive effort evolve independently of offspring size?. Evolution, 55 (6): 1245- 1248. 11475060 10.1111/j.0014-3820.2001.tb00644.x

[11] Clout MN, Elliott GP, Robertson BC. 2002 Effects of supplementary feeding on the offspring sex ratio of kakapo:A dilemma for the conservation of a polygynous parrot. Biological Conservation, 107 (1): 13- 18.

[12] Donelson JM, McCormick MI, Munday PL. 2008 Parental condition affects early life-history of a coral reef fish. Journal of Experimental Marine Biology and Ecology, 360 (2): 109- 116.

[13] Douhard M, Plard F, Gaillard JM, Capron G, Delorme D, Klein F, Duncan P, Loe LE, Bonenfant C. 2014 Fitness consequences of environmental conditions at different life stages in a long-lived vertebrate. Proceedings of the Royal Society B:Biological Sciences, 281 (1785): 20140276- 24789898 10.1098/rspb.2014.0276 PMC4024291

[14] Du WG, Ji X, Shine R. 2005 Does body-volume constrain reproductive output in lizards?. Biology Letters, 1 (1): 98- 100. 17148138 10.1098/rsbl.2004.0268 PMC1629063

[15] Du WG. 2006 Phenotypic plasticity in reproductive traits induced by food availability in a lacertid lizard, Takydromus septentrionalis. Oikos, 112 (2): 363- 369.

[16] Dunham AE. 1978 Food availability as a proximate factor influencing individual growth rates in the iguanid lizard* Sceloporus Merriami*. Ecology, 59 (4): 770- 778.

[17] Einum S, Fleming IA. 1999 Maternal effects of egg size in brown trout (Salmo trutta):norms of reaction to environmental quality. Proceedings of the Royal Society B:Biological Sciences, 266 (1433): 2095- 2100.

[18] Flemming A F, Blackburn D G. 2003 Evolution of placental specializations in viviparous African and South American lizards. Journal of Experimental Zoology Part A, 299A (1): 33- 47. 10.1002/jez.a.10289 12950033

[19] Fox CW, Mousseau TA. 1998 Maternal effects as adaptations for transgenerational phenotypic plasticity in insects.*Maternal Effects as Adaptations*,New York Oxford University Press 159- 177.

[20] Hafer N, Ebil S, Uller T, Pike N. 2011 Transgenerational effects of food availability on age at maturity and reproductive output in an asexual collembolan species. Biology Letters, 7 (5): 755- 758. 21411448 10.1098/rsbl.2011.0139 PMC3169046

[21] Hamilton WD. 1967 Extraordinary sex ratios. Science, 156 (3774): 477- 488. 6021675 10.1126/science.156.3774.477

[22] Hegemann A, Matson KD, Flinks H, Tieleman BI. 2013 Offspring pay sooner, parents pay later:Experimental manipulation of body mass reveals trade-offs between immune function, reproduction and survival. Frontiers in Zoology, 10 77- 24344978 10.1186/1742-9994-10-77 PMC3878409

[23] Hillesheim E, Stearns SC. 1991 The responses of *Drosophila melanogaster* to artificial selection on body weight and its phenotypic plasticity in two larval food environments. Evolution, 45 (8): 1909- 1923. 10.1111/j.1558-5646.1991.tb02696.x 28563960

[24] Hogstedt G. 1981 Effect of additional food on reproductive success in the Magpie (Pica pica). Journal of Animal Ecology, 50 (1): 219- 229.

[25] Hoy SR, Millon A, Petty SJ, Whitfield DP, Lambin X. 2016 Food availability and predation risk, rather than intrinsic attributes, are the main factors shaping the reproductive decisions of a long-lived predator. Journal of Animal Ecology, 85 (4): 892- 902. 26990178 10.1111/1365-2656.12517

[26] Hsu BY, Dijkstra C, Darras VM, de Vries B, Groothuis TGG. 2016 Maternal adjustment or constraint:Differential effects of food availability on maternal deposition of macro-nutrients, steroids and thyroid hormones in rock pigeon eggs. Ecology and Evolution, 6 (2): 397- 411. 26843926 10.1002/ece3.1845 PMC4729257

[27] Iglesias-Carrasco M, Head ML, Cabido C. 2016 Habitat dependent effects of experimental immune challenge on lizard anti-predator responses. Behavioral Ecology and Sociobiology, 70 (11): 1931- 1939.

[28] Irschick DJ, Losos JB. 1998 A comparative analysis of the ecological significance of maximal locomotor performance in Caribbean anolis lizards. Evolution, 52 (1): 219- 226. 10.1111/j.1558-5646.1998.tb05155.x 28568148

[29] Itonaga K, Jones SM, Wapstra E. 2012a. Do gravid females become selfish? Female allocation of energy during gestation. Physiological and Biochemical Zoology, 85 (3): 231- 242. 22494979 10.1086/665567

[30] Itonaga K, Jones SM, Wapstra E. 2012b. Effects of maternal basking and food quantity during gestation provide evidence for the selective advantage of matrotrophy in a viviparous lizard. PLoS One, 7 (7): e41835- 22848629 10.1371/journal.pone.0041835 PMC3406071

[31] Jakob EM, Marshall SD, Uetz GW. 1996 Estimating fitness:A comparison of body condition indices. Oikos, 77 (1): 61- 67.

[32] Johnson JC, Miles LS, Trubl PJ, Hagenmaier A. 2014 Maternal effects on egg investment and offspring performance in black widow spiders. Animal Behaviour, 91 67- 73.

[33] Jones SKC, Munn AJ, Penman TD, Byrne PG. 2015 Long-term changes in food availability mediate the effects of temperature on growth, development and survival in striped marsh frog larvae:Implications for captive breeding programmes. Conservation Physiology, 3 (1): cov029- 27293714 10.1093/conphys/cov029 PMC4778449

[34] Kitaysky AS, Wingfield JC, Piatt JF. 1999 Dynamics of food availability, body condition and physiological stress response in breeding black-legged kittiwakes. Functional Ecology, 13 (5): 577- 584.

[35] Komdeur J, Daan S, Tinbergen J, Mateman C. 1997 Extreme adaptive modification in sex ratio of the Seychelles warbler's eggs. Nature, 385 (6616): 522- 525.

[36] Krist M, Munclinger P. 2015 Context dependence of maternal effects:Testing assumptions of optimal egg size, differential, and sex allocation models. Ecology, 96 (10): 2726- 2736. 26649393 10.1890/14-2450.1

[37] Le Galliard JF, Le Bris M, Clobert J. 2003 Timing of locomotor impairment and shift in thermal preferences during gravidity in a viviparous lizard. Functional Ecology, 17 (6): 877- 885.

[38] Lehman S, Smith AA. 1988 Regional differentiation in the stomach of the green anole. The Anatomical Record, 220 (4): 364- 368. 3382024 10.1002/ar.1092200404

[39] Li H, Ji X, Qu YF, Gao JF, Zhang L. 2006 Sexual dimorphism and female reproduction in the multi-ocellated racerunner (*Eremias multiocellata*) (Lacertidae). Acta Zoologica Sinica, 52 (2): 250- 255.

[40] Ma L, Sun BJ, Li SR, Sha W, Du WG. 2014 Maternal thermal environment induces plastic responses in the reproductive life history of oviparous lizards. Physiological and Biochemical Zoology, 87 (5): 677- 683. 25244379 10.1086/678050

[41] Meserve PL, Vásquez H, Kelt DA, Gutiérrez JR, Milstead WB. 2016 Patterns in arthropod abundance and biomass in the semiarid thorn scrub of Bosque Fray Jorge national park, north-central Chile:A preliminary assessment. Journal of Arid Environments, 126 68- 75.

[42] Neuman-Lee LA, Fokidis HB, Spence AR, Van der Walt M, Smith GD, Durham S, French SS. 2015 Food restriction and chronic stress alter energy use and affect immunity in an infrequent feeder. Functional Ecology, 29 (11): 1453- 1462.

[43] Olsson M, Wapstra E, Olofsson C. 2002 Offspring size-number strategies:Experimental manipulation of offspring size in a viviparous lizard (Lacerta vivipara). Functional Ecology, 16 (1): 135- 140.

[44] Ramírez-Bautista A, Balderas-Valdivia C, Vitt LJ. 2000 Reproductive ecology of the whiptail lizard *Cnemidophorus lineatissimus* (Squamata:Teiidae) in a tropical dry forest. Copeia, 2000 (3): 712- 722.

[45] Ramírez-Pinilla MP. 2006 Placental transfer of nutrients during gestation in an Andean population of the highly matrotrophic lizard genus mabuya (squamata:scincidae). Herpetological Monographs, 20 (1): 194- 204.

[46] Raveh S, Vogt D, Kölliker M. 2016 Maternal programming of offspring in relation to food availability in an insect (*Forficula auricularia*). Proceedings of the Royal Society B:Biological Sciences, 283 (1828): 20152936- 27053749 10.1098/rspb.2015.2936 PMC4843645

[47] Rollinson N, Hutchings JA. 2013 Environmental quality predicts optimal egg size in the wild. The American Naturalist, 182 (1): 76- 90. 10.1086/670648 23778228

[48] Rollinson N, Rowe L. 2016 The positive correlation between maternal size and offspring size:Fitting pieces of a life-history puzzle. Biological Reviews, 91 (4): 1134- 1148. 26289842 10.1111/brv.12214

[49] Rosenfeld CS, Roberts RM. 2004 Maternal diet and other factors affecting offspring sex ratio:A review. Biology of Reproduction, 71 (4): 1063- 1070. 15229140 10.1095/biolreprod.104.030890

[50] Sheldon BC, Verhulst S. 1996 Ecological immunology:Costly parasite defences and trade-offs in evolutionary ecology. Trends in Ecology & Evolution, 11 (8): 317- 321. 21237861 10.1016/0169-5347(96)10039-2

[51] Shine R. 2003 Effects of pregnancy on locomotor performance:An experimental study on lizards. Oecologia, 136 (3): 450- 456. 12750993 10.1007/s00442-003-1281-9

[52] Sinervo B, Licht P. 1991 Proximate constraints on the evolution of egg size, number, and total clutch mass in lizards. Science, 252 (5010): 1300- 1302. 17842955 10.1126/science.252.5010.1300

[53] Smith CC, Fretwell SD. 1974 The optimal balance between size and number of offspring. The American Naturalist, 108 (962): 499- 506.

[54] Styrsky JD, Dobbs RC, Thompson CF. 2000 Food-supplementation does not override the effect of egg mass on fitness-related traits of nestling house wrens. Journal of Animal Ecology, 69 (4): 690- 702.

[55] Sun LX, Shine R, Zhao DB, Tang ZR. 2002 Low costs, high output:Reproduction in an insular pit-viper (*Gloydius shedaoensis*, Viperidae) from north-eastern China. Journal of Zoology, 256 (4): 511- 521.

[56] Tang XL, Yue F, Yan XF, Zhang DJ, Xin Y, Wang C, Chen Q. 2012 Effects of gestation temperature on offspring sex and maternal reproduction in a viviparous lizard (*Eremias multiocellata*) living at high altitude. Journal of Thermal Biology, 37 (6): 438- 444.

[57] Tieleman BI, Dijkstra TH, Klasing KC, Visser GH, Williams JB. 2008 Effects of experimentally increased costs of activity during reproduction on parental investment and self-maintenance in tropical house wrens. Behavioral Ecology, 19 (5): 949- 959.

[58] Trivers RL, Willard DE. 1973 Natural selection of parental ability to vary the sex ratio of offspring. Science, 179 (4068): 90- 92. 4682135 10.1126/science.179.4068.90

[59] Uller T, Isaksson C, Olsson M. 2006 Immune challenge reduces reproductive output and growth in a lizard. Functional Ecology, 20 (5): 873- 879.

[60] Vaissi S, Sharifi M. 2016 Changes in food availability mediate the effects of temperature on growth, metamorphosis and survival in endangered yellow spotted mountain newt:Implications for captive breeding programs. Biologia, 71 (4): 444- 451. 10.1002/zoo.21327 27704614

[61] Vinkler M, Bainová H, Albrecht T. 2010 Functional analysis of the skin-swelling response to phytohaemagglutinin. Functional Ecology, 24 (5): 1081- 1086.

[62] Wang Y, Zeng ZG, Li SR, Bi JH, Du WG. 2016 Low precipitation aggravates the impact of extreme high temperatures on lizard reproduction. Oecologia, 182 (4): 961- 971. 27638182 10.1007/s00442-016-3727-x

[63] Wapstra E, Warner DA. 2010 Sex allocation and sex determination in squamate reptiles. Sexual Development, 4 (1-2): 110- 118. 20051672 10.1159/000272459

[64] Warner DA, Lovern MB, Shine R. 2007 Maternal nutrition affects reproductive output and sex allocation in a lizard with environmental sex determination. Proceedings of the Royal Society B:Biological Sciences, 274 (1611): 883- 890. 17251109 10.1098/rspb.2006.0105 PMC2093968

[65] Warner DA, Lovern MB. 2014 The maternal environment affects offspring viability via an indirect effect of yolk investment on offspring size. Physiological and Biochemical Zoology, 87 (2): 276- 287. 24642545 10.1086/674454

[66] Warner DA, Buckelew AM, Pearson PR, Dhawan A. 2015 The effect of prey availability on offspring survival depends on maternal food resources. Biological Journal of the Linnean Society, 115 (2): 437- 447.

[67] Williams LH. 1966 Observations on the life history of the poplar hawk moth, Laothoe Populi L. Part I. Mating, egg laying and larval development and behaviour. Physiological Entomology, 41 (7-9): 93- 102.

[68] Winne CT, Willson JD, Gibbons JW. 2006 Income breeding allows an aquatic snake *Seminatrix pygaea* to reproduce normally following prolonged drought-induced aestivation. Journal of Animal Ecology, 75 (6): 1352- 1360. 17032367 10.1111/j.1365-2656.2006.01159.x

[69] Zamora-Camacho FJ, Reguera S, Moreno-Rueda G. 2016 Elevational variation in body-temperature response to immune challenge in a lizard. PeerJ, 4 e1972- 27168981 10.7717/peerj.1972 PMC4860334

[70] Zeng ZG, Bi JH, Li SR, Wang Y, Robbins TR, Chen SY, Du WG. 2016 Habitat alteration Influences a desert steppe lizard community:Implications of species-specific preferences and performance. Herpetological Monographs, 30 (1): 34- 48.

[71] Zhu H, Wang DL, Wang L, Fang J, Sun W, Ren BZ. 2014 Effects of altered precipitation on insect community composition and structure in a meadow steppe. Ecological Entomology, 39 (4): 453- 461.

